# Association of root-knot nematode (*Meloidogyne incognita*) infestation and tomato brown rugose fruit virus incidence in greenhouse tomatoes following dimethyl disulfide soil treatment

**DOI:** 10.3389/fpls.2026.1758328

**Published:** 2026-03-09

**Authors:** Muharrem Arap Kamberoglu, Mohamed Hussein

**Affiliations:** 1 Department of Plant Protection, Institute of Natural and Applied Sciences, Cukurova University, Saricam, Adana, Türkiye; 2 Department of Biotechnology, Institute of Natural and Applied Sciences, Cukurova University, Saricam, Adana, Türkiye

**Keywords:** Dimethyl disulfide, *Meloidogyne incognita*, root-knot nematode, soil fumigation, sustainable disease management, tomato brown rugose fruit virus, virus–nematode association

## Abstract

**Introduction:**

Tomato (*Solanum lycopersicum* L.) production is increasingly constrained by root-knot nematodes (RKNs, *Meloidogyne* spp.) and Tomato brown rugose fruit virus (ToBRFV), two destructive pathogens that frequently co-occur in protected cultivation systems. *Meloidogyne incognita* induces severe root galling that weakens plant vigor, while ToBRFV is a highly stable, mechanically transmitted virus responsible for substantial yield losses. This study examined the association between nematode-induced root damage and ToBRFV incidence in tomato using a nematode suppression gradient generated by dimethyl disulfide (DMDS) under field conditions.

**Methods:**

Greenhouse experiments were conducted across two consecutive cropping cycles using replicated greenhouse plots, with all disease and treatment responses evaluated at the plot level. The first crop experienced near-complete failure due to severe RKN infestation accompanied by ToBRFV symptoms. Before establishing the second crop, DMDS was applied at six rates (10–60 g/m^2^) to selectively reduce nematode pressure. Nematode suppression, ToBRFV incidence (determined by RT-PCR), and phytotoxic effects were assessed. Correlation and regression analyses were performed to evaluate the relationship between root damage severity and viral incidence.

**Results:**

DMDS markedly suppressed nematode populations, achieving 94.14% control at 60 g/m². Across the same gradient, plot-level ToBRFV incidence decreased by up to 86.04%, with no phytotoxic effects observed at any application rate. Statistical analyses revealed a strong positive correlation between root rot severity and ToBRFV incidence (Pearson’s r = 0.785, p = 0.0367). Linear modelling showed that root damage explained 61.6% of the variation in ToBRFV incidence (R^2^ = 0.616).

**Discussion:**

These findings provide the first experimental evidence that ToBRFV incidence co-varies with *M. incognita* severity in tomato under greenhouse conditions. The study demonstrates the utility of nematode suppression gradients for disentangling virus–nematode associations and identifies DMDS as an effective soil fumigation strategy for RKN suppression, within which ToBRFV incidence consistently declined along the nematode suppression gradient.

## 1 Introduction

The global demand for tomato (*Solanum lycopersicum* L*.)* continues to rise due to its high nutritional and economic value. Greenhouse cultivation has become a key component in meeting this demand, providing a substantial proportion of total tomato production worldwide (FAOSTAT, 2024; RaboResearch, 2025). While monoculture greenhouse systems maximize yield, they also heighten the risk of soil-borne diseases (Belete and Yadete, 2023). Crop rotation can mitigate disease pressure and improve soil health, but it is often impractical in intensive production systems (Jiang et al., 2022). Consequently, tomato crops grown under such systems are highly vulnerable to multiple biotic stresses, particularly plant-parasitic nematodes (PPNs), which severely reduce yield and fruit quality (Nicol et al., 2011).

Among PPNs, root-knot nematodes (*Meloidogyne* spp.) are considered the most destructive, inducing characteristic root galls and yield losses ranging from 42% to 54% in tomato (Netscher and Sikora, 1990; Sikora and Fernandez, 2005; Devran and Söğüt, 2011; Sharaf et al., 2016; Niu et al., 2020). To date, 98 *Meloidogyne* species have been reported, infecting over 3,000 plant species (Hunt and Handoo, 2009; Jones et al., 2013). In Türkiye, RKNs are prevalent across the Mediterranean, Aegean, and Black Sea regions, with dominant species including *M. incognita*, *M. arenaria*, *M. chitwoodi*, *M. exigua*, *M. hapla*, *M. javanica*, and *M. thamesi* (Yüksel, 1966, Yüksel, 1967; Elekcioglu, 1992; Kepenekci, 2002; Ozarslandan et al., 2009; Aydınlı et al., 2013; İmren et al., 2014). Other species such as *M. artiellia* and *M. luci* have also been detected in cereals and solanaceous crops (Aydınlı et al., 2013; İmren et al., 2014). Because crop rotation is rarely feasible in greenhouse production, chemical control has historically been the primary management strategy for RKNs (Zasada et al., 2010; Hajihassani, 2018). However, increasing environmental concerns and regulatory restrictions are driving the search for more sustainable alternatives (Baidoo et al., 2017; Forghani and Hajihassani, 2020).

In parallel with nematode pressure, viral pathogens—particularly Tomato brown rugose fruit virus (ToBRFV; genus Tobamovirus)—pose a serious threat to tomato production. ToBRFV was first reported in greenhouse-grown tomatoes in Jordan in 2015 (Salem et al., 2016) and spreads efficiently through mechanical contact involving contaminated tools, hands, clothing, and surfaces (Salem et al., 2020; Panno et al., 2020). The virus exhibits remarkable environmental persistence, remaining infectious in soil and plant debris and thereby serving as long-term inoculum source (Luria et al., 2017; Oladokun et al., 2019). Infectivity can persist even in soil and soilless substrates amended with infected debris, as biosolarization and biofumigation treatments up to 60 days have been shown to be insufficient to prevent infection of subsequent host plants (Janssen et al., 2025). Furthermore, ToBRFV has been shown to remain infectious in irrigation and drainage water for up to four weeks at room temperature, and irrigation with contaminated water can result in plant infection, highlighting water as an additional pathway for virus dissemination in greenhouse systems (Mehle et al., 2023). ToBRFV has been demonstrated to localize primarily in the seed coat and, less frequently, in the endosperm, but not in the embryo, with low yet epidemiologically relevant seed transmission rates (Davino et al., 2020). ToBRFV, as a tobamovirus, is not truly seed-transmitted via embryo infection but is introduced at low frequency through mechanical inoculation when emerging seedlings contact virus-contaminated seed coats. Despite this limited primary transmission, even minimal initial inoculum can initiate severe epidemics in greenhouse systems due to the highly efficient mechanical spread characteristic of tobamoviruses (Oladokun et al., 2019). Although thermal and chemical disinfection treatments can effectively inactivate infectivity, viral RNA often remains detectable by RT-qPCR, thereby complicating phytosanitary diagnostics and seed health certification procedures (Davino et al., 2020). Genetic resistance is a cornerstone of viral disease management, yet ToBRFV is capable of overcoming all known tobamovirus resistance genes, including Tm-2^2^ (Luria et al., 2017). The breakdown of resistance has intensified reliance on strict hygiene protocols and chemical disinfectants to reduce mechanical transmission. Various disinfectants have been evaluated for their efficacy against ToBRFV and related tobamoviruses, including chlorine dioxide (ClO_2_), chlorinated trisodium phosphate (TSP-Cl), sodium hypochlorite, hydrogen peroxide-based formulations, and quaternary ammonium compounds (Gutiérrez et al., 2024; Mani et al., 2024). A comprehensive table summarizing these disinfectants and their application scenarios is provided in the review by Salem et al., 2023. While these measures can reduce surface contamination and limit secondary spread, they do not eliminate soilborne inoculum nor address factors that may predispose plants to infection. Consequently, effective control remains challenging in intensive greenhouse systems due to the high environmental stability and efficient mechanical transmission of the virus.

Sulfur-containing compounds play critical roles in plant defense against a broad range of pathogens (Bloem et al., 2005; Nwachukwu et al., 2012). Extracts from garlic (*Allium sativum*) exhibit broad-spectrum antiviral activity against several plant viruses, including ToBRFV (Rouf et al., 2020; Iobbi et al., 2023). Dimethyl disulfide (DMDS), a sulfur-containing compound of plant and microbial origin, demonstrates nematicidal, fungicidal, bactericidal, and insecticidal properties (Coley-Smith, 1986; Huang et al., 2012; Meldau et al., 2013; Eure and Culpepper, 2017; Zhong et al., 2021; Awasthi et al., 2025; Shi et al., 2025). Under greenhouse conditions, DMDS is an effective soil fumigant against RKNs and is considered environmentally safer than many conventional nematicides (Auger et al., 1989; Heller et al., 2009; Wang et al., 2011; Yan et al., 2019). In addition, recent evidence suggests that DMDS may function as a signaling molecule capable of priming plant defenses against insect pests (Wang et al., 2025). Given that other chemical inducers such as ISR2000 and harpin-protein formulations can activate antiviral responses (Hussein and Kamberoğlu, 2017a, 2017b), DMDS may similarly contribute to enhanced resistance against viral infection, although no direct antiviral activity has been demonstrated to date and such effects remain to be experimentally verified. When applied as a soil drench, DMDS has demonstrated antimicrobial activity and the potential to elicit induced systemic resistance in tobacco and corn against fungal pathogens under greenhouse conditions (Huang et al., 2012).

To better understand the antimicrobial and plant-mediated effects of DMDS, it is essential to consider its natural occurrence and inherent mode of action in the environment. DMDS is a naturally occurring sulfur-containing compound found in *Allium* species as well as members of the Brassicaceae family (e.g., cabbage, radish, canola), where it forms part of the plant chemical defense arsenal (Wang et al., 2009; Arnault et al., 2013). Upon tissue disruption, sulfur volatiles are released through the hydrolysis of S-methyl-L-cysteine sulfoxide (SMCSO), generating volatile organosulfur compounds (VOSCs) such as methyl sulfide (MS) and DMDS (Horníčková et al., 2009; Andernach et al., 2024). These volatiles contribute to natural protection against soil-borne pathogens by disrupting microbial membrane integrity and inducing cell wall damage and ultrastructural alterations (Tyagi et al., 2020), ultimately leading to leakage of cellular contents and cell death. Studies on related sulfur compounds, such as dimethyl trisulfide (DMTS), indicate that pathogen suppression may involve impairment of mitochondrial function and modulation of stress-related enzymes, suggesting a similar mode of action for DMDS (Auger and Charles, 2003; Qu et al., 2025).

In addition to direct toxicity, DMDS has been shown to inhibit spore development, including suppression of sclerotia formation and germ tube emergence in *Sclerotinia minor* (Tyagi et al., 2020), and inhibition of conidial germination in *Aspergillus flavus* by DMDS-rich bacterial volatiles (Gong et al., 2019). DMDS can also induce oxidative stress in pathogens by promoting excessive production of reactive oxygen species (ROS), including hydrogen peroxide accumulation, thereby disrupting cellular homeostasis (Tyagi et al., 2020; Jiang et al., 2025). Although direct evidence of DMDS-induced DNA damage is limited, ROS generation during exposure may contribute to DNA lesions (Yu and Anderson, 1997). Sulfur-containing bio-compounds are known to induce ROS-mediated DNA damage in eukaryotic cells (Hayatsu and Miller, 1972; Mathan et al., 2024; Xia et al., 2024) and to trigger sulfur-enhanced defense (SED) or sulfur-induced resistance (SIR) in plants, particularly against soil-borne fungal pathogens (Künstler et al., 2020). The disulfide reactivity of DMDS allows covalent modification of thiol groups in microbial enzymes, leading to enzyme inactivation, through thiol–disulfide exchange reactions (Nwachukwu et al., 2012; Chinchilla et al., 2019).

Beyond its direct antimicrobial activity, DMDS can also enhance plant immunity by activating salicylic acid–dependent pathways and inducing pathogenesis-related (PR) gene expression (Meldau et al., 2013; Liu et al., 2020; Tyagi et al., 2020; Ling et al., 2022). Many root-associated bacteria, such as *Bacillus* spp. and *Pseudomonas* spp., produce DMDS and other VOSCs as secondary metabolites (Meldau et al., 2013; Zhong et al., 2021). These microbial volatiles exhibit strong antipathogenic activity and can trigger induced systemic resistance (ISR) in plants through salicylic acid–mediated signaling (Ryu et al., 2004).

In nature, plants are frequently exposed to simultaneous infections by multiple pathogens, resulting in complex interactions that can be antagonistic, synergistic, or neutral (Abdullah et al., 2017; Dutt et al., 2022). Some studies have reported reduced nematode populations in virus-infected plants (Youssef et al., 2011; Azaryan et al., 2024), while others observed enhanced nematode penetration in virus-infected hosts (Bird, 1969; Ryder and Crittenden, 1962; Swarup and Goswami, 1969). These interactions can significantly alter disease severity, yet their underlying physiological and biochemical mechanisms remain poorly understood (Weischer, 1975; Roy et al., 2025).

Despite extensive research on virus–nematode associations, no studies have specifically explored the interaction between ToBRFV and RKNs. This study therefore evaluated the natural co-occurrence of ToBRFV and *Meloidogyne incognita* in greenhouse-grown tomato and assessed whether ToBRFV incidence co-varied with nematode suppression achieved through graded applications of DMDS. The objective was to clarify associative patterns relevant to integrated and sustainable disease management in intensive greenhouse tomato production.

## 2 Materials and methods

### 2.1 Experimental site and design

The field experiment was conducted in a commercial tomato (*Solanum lycopersicum* L.) greenhouse located in Mersin Province, Türkiye (36°56′42.55″ N, 29°55′34.12″ E), with a documented history of severe root-knot nematode (RKN; *Meloidogyne* spp.) infestation and ToBRFV infection. Plants exhibited extensive root galling with an estimated survival rate of 7.88%, and ToBRFV presence was confirmed by laboratory diagnostics. To examine whether viral incidence co-varied with nematode suppression, infestation levels were reduced using graded applications of DMDS.

The study relied on the naturally co-occurrence of *M. incognita* and ToBRFV; no inoculum was experimentally introduced. Pre-treatment assessments showed an average initial RKN density of 150 second-stage juveniles (J2) per 100 g of soil, a population level previously characterized in tomato pathogenesis studies (Bozbuga et al., 2015). PCR analysis of residual root debris from all plots prior to treatment confirmed uniform baseline ToBRFV distribution across the site.

**Figure 1:**
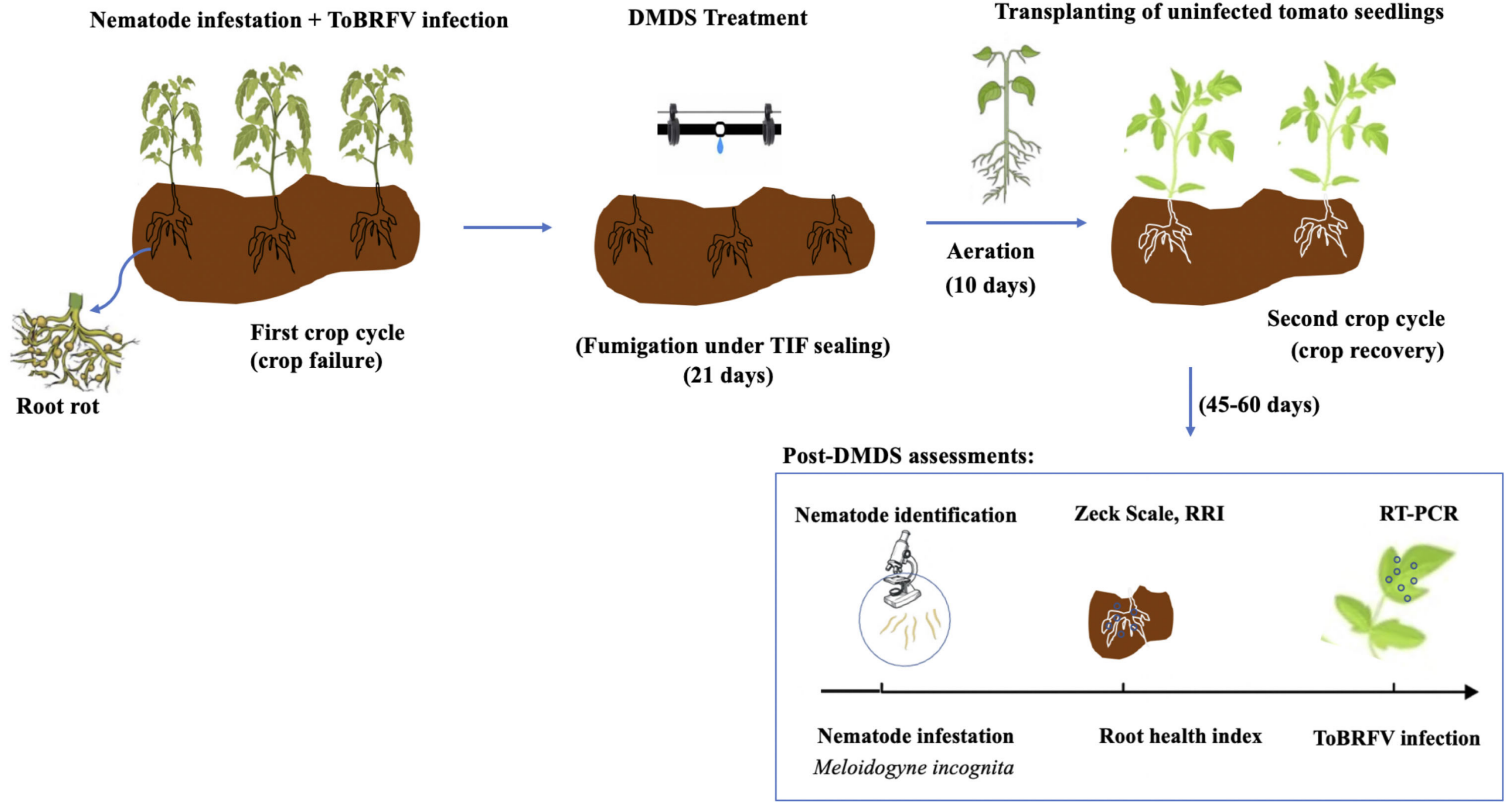
Experimental design and post-DMDS assessments.During the first crop cycle, tomato plants were heavily infested with *Meloidogyne incognita* and infected with ToBRFV. In the second crop cycle, the infested soil was fumigated with different doses of DMDS under TIF sealing for 21 days, after which uninfected tomato seedlings were transplanted, with 10 days of aeration, and subsequently assessed for nematode infestation (Zeck scale and root health index) and for ToBRFV infection (RT–PCR).

The greenhouse was divided into experimental plots arranged in a randomized complete block design, with five replicates per treatment. Each plot constituted a treatment unit measuring 8 × 5 m (40 m²) and was separated by buffer zones of at least 1.5 m. Six DMDS application rates (10–60 g/m²) and an untreated control were applied to establish a gradient of nematode suppression. All plots were managed using identical standard commercial greenhouse practices (**Figure 1**).

Measures to limit plot-to-plot interference included buffer zones, sequential sampling, and the disinfection or replacement of gloves, clothing, and tools between plots, thereby minimizing the risk of mechanical virus transmission and unintended movement of nematodes or DMDS residues among treatment levels.

### 2.2 Soil preparation and fumigation

The soil at the experimental site was medium-textured (sandy loam to clay loam) with 25–30% clay. Soil pH ranged from 6.5 to 7.8, electrical conductivity from 1.2 to 2.5 dS/m, and organic matter content from 1.5 to 3.0%. Total nitrogen was 0.08–0.15%, available phosphorus (P_2_O_5_) 25–45 mg/kg, exchangeable potassium (K_2_O) 180–250 mg/kg, and CaCO_3_ content 8–15%.

Before the trial, soil moisture was maintained at 18–25% using drip irrigation. Initial root-knot nematode populations ranged from 120 to 180 J2 per 100 g of soil. No fumigation was carried out prior to DMDS application.

Plant debris and crop residues were removed as part of standard pre-fumigation sanitation procedures, and the topsoil was tilled to a depth of 30 cm to ensure uniform soil structure and aeration. Drip irrigation lines were installed in each plot for controlled application of the fumigant solution. Plots were then covered with 25 µm Totally Impermeable Film (TIF) to reduce gas loss and improve fumigant retention.

DMDS was applied as a ready-to-use emulsifiable concentrate (EC) containing 94% DMDS (technical grade, purity 99.2%), 5% emulsifier, and 1% odorant. The product was used as supplied by the manufacturer and was not modified on site. It was diluted directly in irrigation water to obtain the target application rates (10–60 g/m²).

All application rates are expressed as g/m² in the manuscript. In some sections, rates were initially given in L/da; these values are approximately equivalent because the density of DMDS is close to 1 g/mL. The applied dose was always calculated on an area basis (g/m²). Irrigation water was used only as a carrier to distribute the product and did not change the applied dose. The irrigation volume (20,000 L/da) was the same for all treatments to ensure even distribution in the soil.

DMDS was applied through a calibrated drip chemigation system using 20,000 L water per decare (about 800 L per 40 m² plot), which is consistent with standard greenhouse fumigation practice. Flow rate, pressure, and irrigation volume were the same in all plots. Soil temperature at 20 cm depth during application averaged 26.9 °C. After application, the greenhouse was sealed for 21 days to retain the fumigant in the soil.

### 2.3 Planting and monitoring

Twenty days after fumigation, TIF covers were removed, and the soil was aerated for 10 days before transplanting tomato seedlings (*S. lycopersicum* cv. Dilara). They were planted in a double-row arrangement at a density of 50 plants per 40 m² plot. Plants were monitored throughout the growing period. Phytotoxicity was visually assessed based on leaf color, growth rate, and morphological abnormalities.

### 2.4 Nematode assessment

Nematode infestation was evaluated 45–60 days after planting using 20 randomly selected plants per plot. Root galling severity was scored according to the Zeck (1971) scale (1–10), where 1 = no galls, 2–3 = trace galls, 4–5 = moderate galls, 6–7 = severe galls, 8–10 = very severe galls. Nematode species were initially identified microscopically based on morphological characteristics using standard taxonomic keys (Southey, 1986).

Root rot severity was summarized using a Root Rot Index (RRI), calculated as a disease severity index (DSI) based on ordinal ratings. The DSI concept follows the infection index proposed by Mckinney (1923) and later described by Chester (1950) and is widely used in plant pathology (Chaube and Singh, 1991; Bock et al., 2022; Chiang and Bock, 2022). The RRI expresses overall disease severity as a percentage value for each plot. Individual plants were rated on a 0–10 severity scale, and the RRI (Equation 1) was calculated as:

(1) $$RRI=\;\frac{\sum \left(n_{i}\times s_{i}\right)}{N\;\times S_{max}}\times 100$$

where *_n_* is the number of plants at severity score *_s_*, *N* is the total number of plants evaluated, and *S_max_* is the maximum severity score.

The Effectiveness (Equation 2) of DMDS in controlling root rot was expressed as disease recovery (%) using Abbott’s formula:

(2) $$Effectiveness\left(\%\right)=\frac{RRI_{control}-RRI_{treatment}}{RRI_{control}}\times 100$$

A value of 0% indicated no recovery (maximum symptom severity), and 100% indicated complete recovery (no symptoms).

### 2.5 Total nucleic acid extraction

Total nucleic acids (TNA) were extracted from fresh tomato leaves following the protocol of Astruc et al. (1996). Approximately 0.5 g of leaf tissue was homogenized in extraction buffer (100 mM Tris-HCl pH 8.0, 50 mM EDTA, 500 mM NaCl, 0.1% 2-mercaptoethanol) and treated with 50 µL of 20% SDS. After centrifugation, 250 µL of 6 M potassium acetate (pH 6.5) was added, followed by incubation at 65 °C for 15 min and cooling on ice for 20 min. Nucleic acids were precipitated with ethanol, air-dried, and resuspended in 50 µL RNase-free water. TNA concentration and purity were determined spectrophotometrically, and samples were stored at –80 °C until RT-PCR analysis.

### 2.6 RT–PCR detection of ToBRFV

Complementary DNA (cDNA) was synthesized from 2 µL of TNA using a ToBRFV-specific reverse primer (5′-CGA ATG TGA TTT AAA ACT GTG AAT-3′). The mixture was heated at 70 °C for 2 min, chilled on ice, and reverse-transcribed at 42 °C for 60 min using MMLV reverse transcriptase in the presence of RNase inhibitor, dNTPs, and 1× MMLV buffer. PCR amplification was performed using 2 µL of cDNA, ToBRFV-specific primer pairs (Forward: 5′-AAT GTC CAT GTT TGT TAC GCC-3′; Reverse: 5′-CGA ATG TGA TTT AAA ACT GTG AAT-3′), Taq DNA polymerase, and 1× Taq buffer to amplify a 560-nt fragment of the replicase subunit (GenBank accession no. MK165457; Alkowni et al., 2019). The cycling conditions were: 94 °C for 2 min; 35 cycles of 94 °C for 30 s, 53 °C for 30 s, 72 °C for 2 min; and a final extension at 72 °C for 10 min. PCR products were separated on 2% agarose gels and visualized under UV light.

ToBRFV incidence was determined by RT-PCR analysis of 10 randomly selected plants per plot (≈20%). This subsampling intensity was considered sufficient to represent plot-level infection status. The number of ToBRFV-positive plants was recorded and expressed as a percentage of the total tested. Incidence (%) (Equation 3) was calculated as:

(3) $$Incidence\left(\%\right)=\frac{Number\;of\;infected\;plants}{Total\;number\;of\;plants\;assessed}\times 100$$

Incidence values corresponding to 0–100% infection (0/10 to 10/10 plants positive) were used to summarize plot-level virus presence and to compare ToBRFV incidence among DMDS-treated and untreated plots, rather than to estimate field-wide prevalence or virus transmission beyond the experimental units.

### 2.7 Statistical analysis

All experiments were arranged in a completely randomized design with five replicate plots per treatment. The experimental plot was treated as the statistical unit. Root galling index (GI) and RRI were calculated from assessments of 20 randomly selected plants per plot, while ToBRFV incidence was determined from 10 randomly selected plants per plot, and plot-level means were used for analysis. Accordingly, all associations reported in this study are based on plot-level indices derived from independent plant subsamples rather than paired measurements on the same plants. The effects of DMDS dose on GI, RRI, and ToBRFV incidence were analyzed using one-way analysis of variance (ANOVA). When significant differences were detected (p< 0.05), Tukey’s Honest Significant Difference (HSD) test was applied for pairwise comparisons among treatments, and statistically distinct groups were indicated by letters in the corresponding figures and tables.

Pearson’s product–moment correlation coefficient was used to evaluate the plot-level association between RRI and ToBRFV incidence across DMDS treatments after verifying parametric assumptions. A simple linear regression model was subsequently applied to describe the relationship between plot-level RRI and virus incidence. Regression coefficients, standard errors (SE), t-values, p-values, and coefficient of determination, R²) were reported. All statistical analyses and graphical outputs were performed in R software (version 4.5.1; R Core Team, 2025) using the stats and ggplot2 packages, and results are presented as mean ± SE.

## 3 Results

### 3.1 Nematode infestation and root health

**Figure 2:**
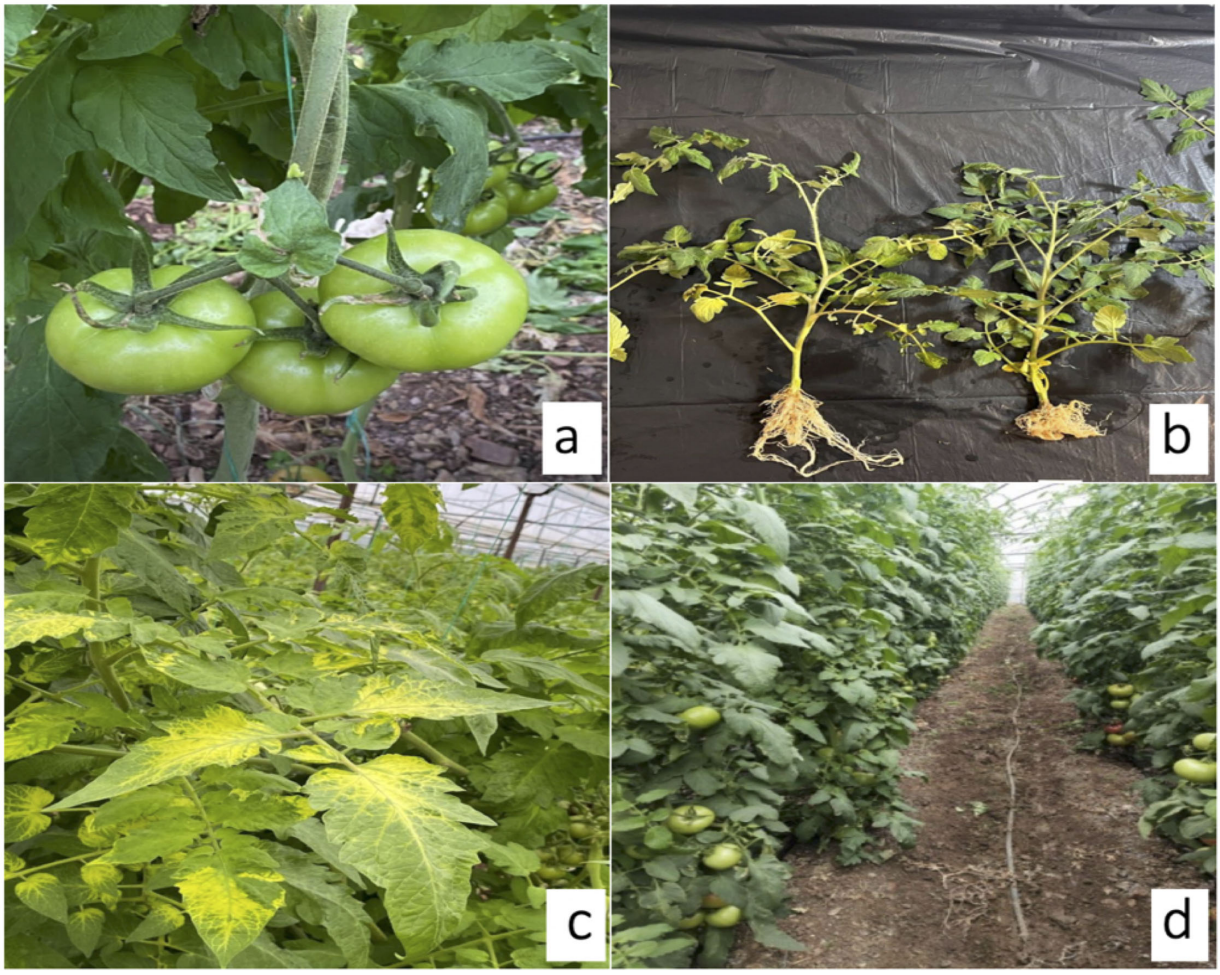
Plant responses to nematode infestation, viral infection, and DMDS treatment. **(a)** non-phytotoxic effects observed at 60 g/m² DMDS; **(b)** plants infested by *Meloidogyne incognita*; **(c)** plants with ToBRFV symptoms; **(d)** DMDS-treated plants (60 g/m²) showing improved health.

**Figure 3:**
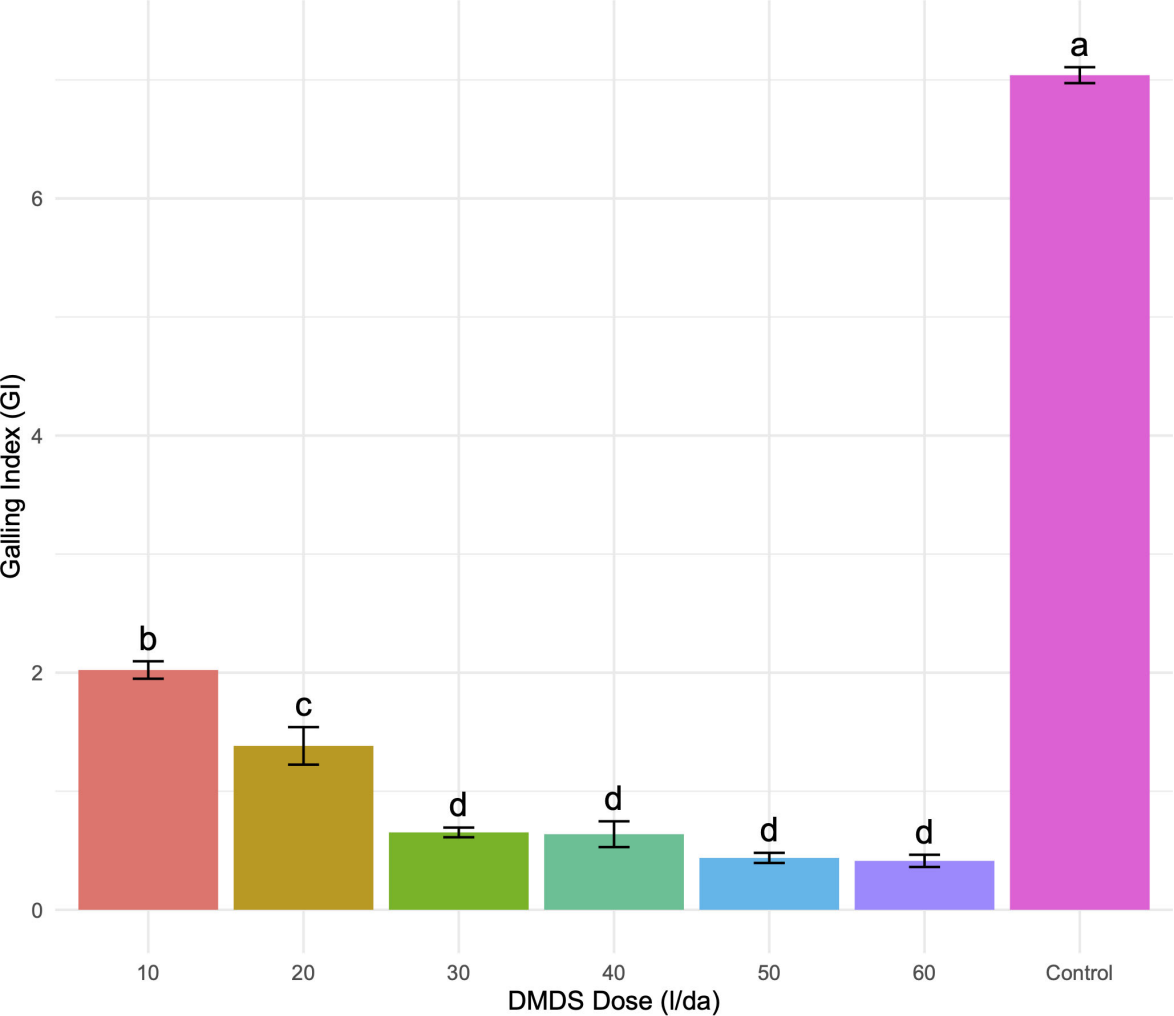
Effect of DMDS dose on root-knot nematode galling index (GI) in tomato. Tomato plants were treated with different doses of DMDS (10, 20, 30, 40, 50, 60 l/da) or left untreated (control). Bars represent mean GI ± SE for four replicates per treatment (n = 5). Different letters above bars indicate significant differences among treatments according to Tukey’s HSD *post-hoc* test, α = 0.05. One-way ANOVA showed a significant effect of DMDS dose on galling index (F_6,21_ = 751.73, p< 0.001). High DMDS doses (50–60 l/da) effectively suppressed root galling compared to control and lower doses, demonstrating a dose-dependent nematicidal activity. DMDS rates in this figure are expressed as L/da; these values are directly equivalent to g/m2 based on a DMDS density of one.

Root-knot nematodes in the experimental plots were confirmed as *Meloidogyne incognita* through morphological identification. Infested plants showed severe galling and extensive root deformation (**Figure 2b**) DMDS application markedly reduced nematode damage in a dose-dependent manner. Severe galling persisted at 10–20 g/m² (Zeck 5–8), declined to moderate at 30 g/m² (index 4–5), and was strongly suppressed at 50 g/m² (2–3). At 60 g/m², galling was minimal (index 1) (**Figure 3**). GI differed significantly among treatments (ANOVA, p< 0.001), and Tukey’s HSD grouped 50–60 g/m² as the most effective doses, clearly separated from lower treatments and the control. Control plants exhibited the highest GI (~7.0), while intermediate doses (20–40 g/m²) provided partial suppression, confirming a strong dose-dependent nematode control pattern.

### 3.2 Root rot severity

Root rot severity increased in the presence of *M. incognita*, likely due to nematode-induced entry points facilitating secondary infections. DMDS significantly reduced the root rot index (RRI) across doses, with the strongest suppression at 60 g/m² (RRI = 5.86 ± 0.99%; Effectiveness = 94.14 ± 0.85%, **Table 1**). A significant dose effect was detected (ANOVA, p< 0.001), and Tukey’s HSD indicated that doses ≥30 g/m² produced significantly higher Effectiveness and lower RRI than the control and lower doses. The untreated control had the highest RRI (100%) and no suppression. These results indicate that effective nematode control by DMDS also mitigates subsequent root-rot development by preserving root structure.

**Table 1:** Dose-dependent effects of DMDS soil application on root rot suppression and ToBRFV incidence in tomato plants.

DMDS dose (g/m²)	Effectiveness (%) Mean ± SE (n=20, 5 reps)	RRI (%) Mean ± SE (n=20, 5 reps)	ToBRFV Incidence (%) Mean ± SE (n=10, 5 reps)
10	71.27 ± 1.44^a^	28.73 ± 2.42^a^	78 ± 3.74^a^
20	80.36 ± 2.94^b^	19.64 ± 3.57^b^	58 ± 3.74^b^
30	90.73 ± 1.28^c^	9.27 ± 1.54^c^	38 ± 3.74^c^
40	90.94 ± 2.11^c^	9.06 ± 2.26^c^	28 ± 3.74^cd^
50	93.79 ± 1.49^d^	6.21 ± 1.02^d^	18 ± 3.74^de^
60	94.14 ± 0.85^d^;	5.86 ± 0.99^d^	12 ± 2.00^e^
Control	0.00 ± 0.00^e^	100.00 ± 0.00^e^	86 ± 2.45^a^

Values represent plot-level means ± SE based on five biological replicate plots per treatment, with the experimental plot treated as the statistical unit. Root rot index (RRI) and effectiveness (%) were calculated from assessments of 20 randomly selected plants per plot. Effectiveness (%) represents the relative reduction in root rot severity in DMDS-treated plots compared with the untreated control, calculated from RRI values. ToBRFV incidence (%) was determined from 10 randomly selected plants per plot. One-way ANOVA indicated a significant effect of DMDS dose on all parameters (p< 0.001). Differences among treatments were evaluated using Tukey’s HSD test (p< 0.05); different superscript letters indicate statistically significant differences among doses.

### 3.3 ToBRFV infection following nematode damage

**Figure 4:**
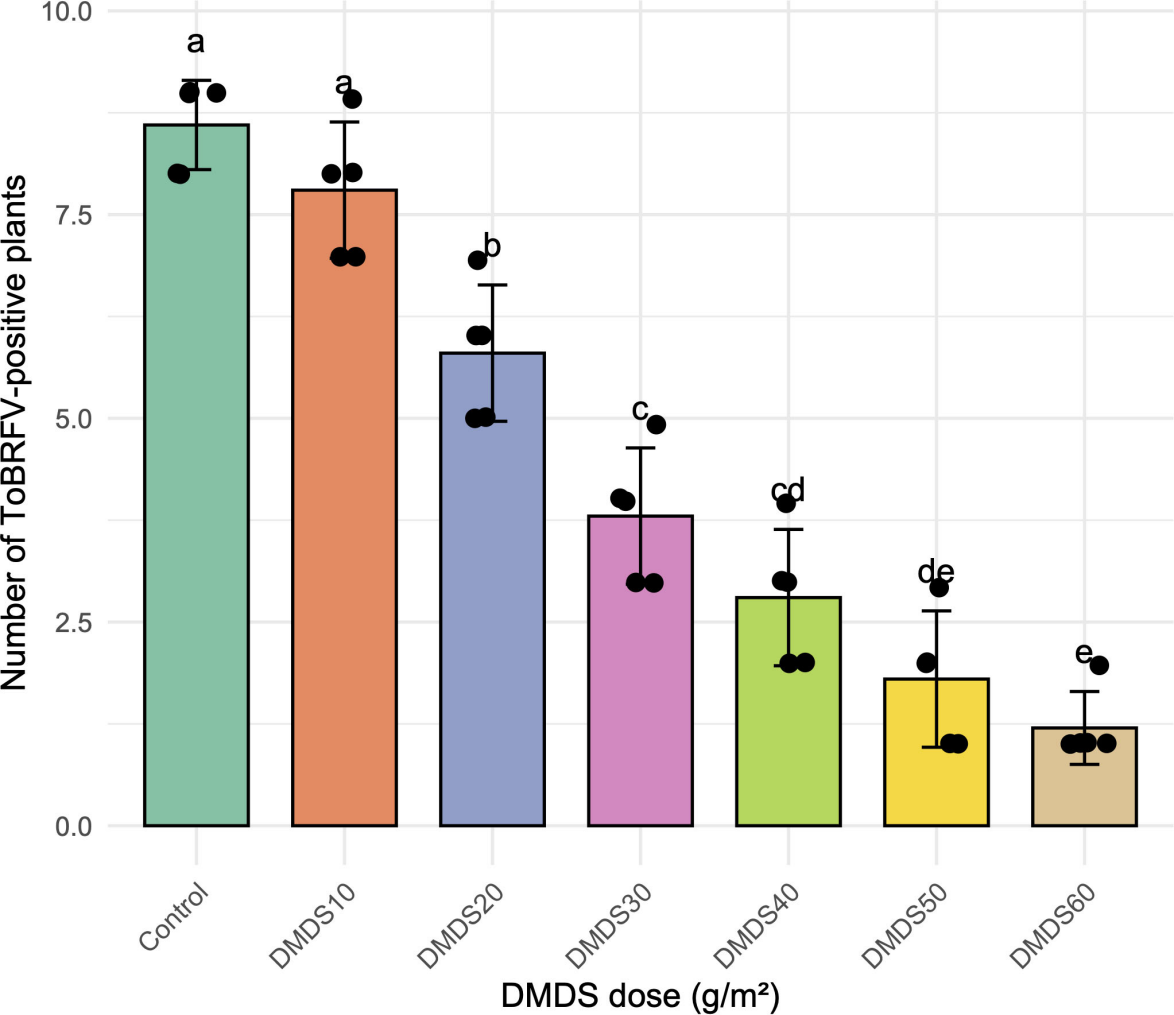
Effect of DMDS on ToBRFV virus incidence in tomato plants.Virus-positive plants were counted per replicate (n = 5 replicates of 10 plants each) following DMDS application at doses of 10–60g/m^2^, compared to an untreated control. Bars represent the mean number of virus-positive plants ± standard deviation, and black dots indicate individual replicate values. Letters above the bars denote statistically significant differences between treatments (Turkey's HSD test, p< 0.05). Higher DMDS doses (30g/m^2^) significantly reduced viral incidence, with 60g/m^2^ achieving near-complete suppression.

ToBRFV symptoms appeared following nematode-induced root stress (**Figure 2c**). Plot-level RT-PCR screening of ten plants per replicate showed a progressive reduction in the number of virus-positive plants across plots exhibiting lower root-knot nematode pressure (**Figure 4**; **Table 1**). Control plots showed the highest ToBRFV incidence (8.6 ± 0.55 virus-positive plants). Plots with partial nematode suppression showed intermediate levels of viral detection (7.8 ± 0.84 and 5.8 ± 0.84 virus-positive plants), whereas plots with stronger nematode suppression exhibited substantially fewer infected plants, with virus incidence reduced to less than half of control levels. The lowest ToBRFV incidence was observed in plots with the greatest nematode suppression (1.2 ± 0.4 virus-positive plants; **Figure 2d**). ANOVA (p< 0.001) followed by Tukey’s HSD (p< 0.05) identified plots with higher levels of nematode suppression as significantly different in ToBRFV incidence from the control and from plots with weaker suppression (**Figure 4**).

### 3.4 Association between root rot severity and ToBRFV incidence

**Figure 5:**
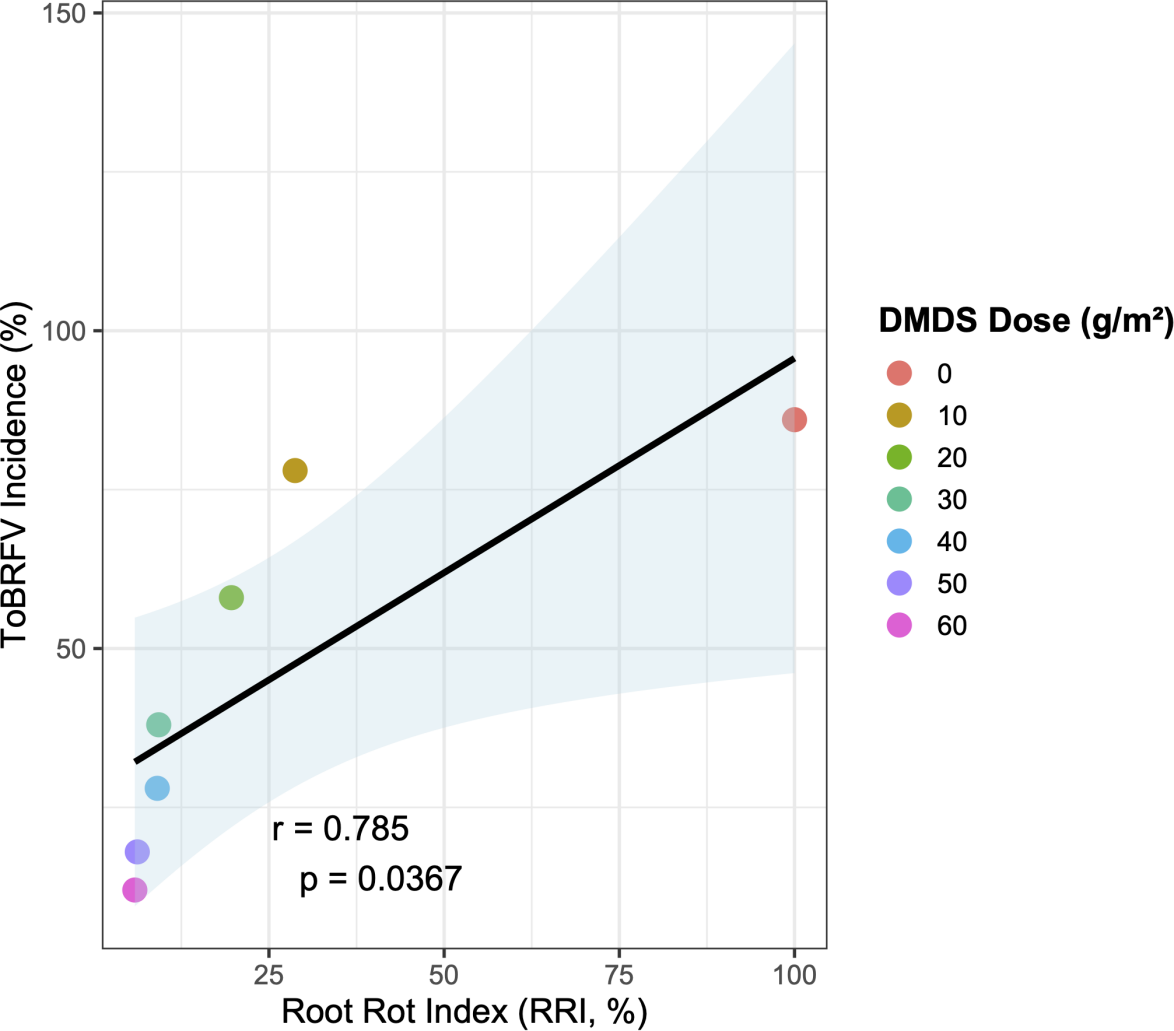
Association between nematode-induced root rot severity (RRI) and ToBRFV incidence in tomato plants under different DMDS treatment levels. The relationship between the root rot index (RRI, %) and ToBRFV incidence (%) was evaluated across different DMDS application rates (0–60 g/m²). Each data point represents the treatment mean (n = 5 replicates for RRI; n = 5 replicates for ToBRFV incidence). A linear regression line was fitted, with the shaded area representing the 95% confidence interval around the fitted line. The Pearson correlation coefficient (r) and the corresponding p-value are displayed on the plot. Correlation analysis revealed a significant positive association between RRI and ToBRFV incidence (r = 0.785, p = 0.0367), with RRI accounting for 61.6% of the variation in viral incidence (R² = 0.616). The regression slope (+0.6748) indicates that, on average, a 1% increase in RRI was associated with an approximately 0.67% increase in ToBRFV incidence. Increasing DMDS application rates were associated with concurrent reductions in both nematode-induced root damage and ToBRFV incidence. These results suggest that nematode infestation severity is closely associated with ToBRFV occurrence, supporting a potential interaction between root-knot nematode damage and viral infection under greenhouse conditions.

To examine the association between root damage and viral occurrence, the relationship between the RRI and ToBRFV incidence was analyzed across DMDS treatments (0–60 g/m²). A strong positive correlation was detected (Pearson’s r = 0.785, p = 0.0367), indicating that plots with higher RRI values consistently exhibited higher ToBRFV incidence (**Figure 5**). Simple linear regression showed that RRI was a significant statistical predictor of ToBRFV incidence (β = 0.67 ± 0.24 SE, t = 2.83, p = 0.036), accounting for 61.6% of the observed variance (R² = 0.616). Higher levels of root damage were associated with proportionally greater levels of viral detection across treatments. The untreated control (RRI = 100%) showed among the highest ToBRFV incidence, further supporting this pattern of co-occurrence. These results demonstrate a strong association between nematode-related root damage and ToBRFV incidence under naturally occurring disease pressure, without implying a direct causal relationship.

### 3.5 Phytotoxicity observations

No phytotoxic symptoms were observed at any DMDS dose, including 60 g/m². Plant growth, leaf morphology, and overall development remained comparable to untreated controls (**Figure 2a**).

## 4 Discussion

### 4.1 Impact of root-knot nematodes on tomato health and viral susceptibility

Under the naturally occurring disease pressure examined in this study, *M. incognita* infestation was associated with pronounced root damage that co-occurred with higher ToBRFV incidence across plots. This pattern supports a strong association between nematode-induced root injury and viral occurrence, without implying direct causation. Both nematode infestation and viral incidence may respond independently to shared environmental or host-related stress factors, and pre-existing viral infection may also influence host physiology in ways that alter plant susceptibility to nematode infestation. Accordingly, the observed patterns are interpreted as correlative associations under field conditions rather than direct cause-and-effect relationships. Notably, virus incidence varied with nematode infestation intensity rather than being uniformly distributed across the greenhouse. Although this does not exclude the influence of shared environmental factors, it suggests that localized processes may contribute to the observed association.

Root-knot nematodes are known to impair water and nutrient uptake, disrupt root architecture, and alter host physiological and defense responses, thereby predisposing plants to secondary pathogens (Powell, 1971; Pulavarty et al., 2021; Lopez-Nicora et al., 2025). The co-occurrence of nematodes and viruses is often described as a disease complex, although such interactions are frequently indirect and context dependent. Here, the term “disease complex” is used strictly to denote the repeated association of nematode infestation and viral infection under field conditions, rather than a direct mechanistic linkage.

Importantly, *M. incognita* is not a vector of ToBRFV. Unlike nematode species that directly transmit soil-borne viruses (e.g., nepoviruses or tobraviruses; Karanastasi and Brown, 2004; Bird and Yan, 2025; Chatterjee et al., 2025; Roy et al., 2025), the association observed in this study appears to be mediated by nematode-induced plant stress rather than direct virus transmission.

Two non-mutually exclusive biological mechanisms may plausibly explain the observed association. First, local effects of root damage may play a role. Extensive galling and tissue disruption caused by nematode feeding can create physical discontinuities in root tissues that may facilitate viral entry or systemic movement from roots to aerial tissues. Experimental root-wounding and immersion studies have demonstrated that damaged roots can serve as effective entry points for systemic viral spread (Li et al., 2024), supporting this possibility under greenhouse conditions.

Second, systemic physiological effects of nematode infection may indirectly increase viral susceptibility. Nematode-induced root damage has been shown to alter host physiology beyond feeding sites, increasing susceptibility to secondary pathogens at the whole-plant level (Powell, 1971). More recent studies have shown that nematode-secreted effectors, including fatty acid– and retinol-binding (FAR) proteins, suppress jasmonic acid–dependent defense signaling pathways that are central to plant resistance responses (Iberkleid et al., 2013; Goverse and Smant, 2014). Such suppression may indirectly compromise antiviral defense capacity, allowing more efficient viral establishment in plants experiencing severe nematode stress.

Additionally, nematodes can influence soil microbial composition, potentially reducing populations of beneficial bacteria that contribute to soil health (Kudjordjie et al., 2024; van Sluijs et al., 2025). Because healthier soil microbiomes can suppress viral incidence in tomato (Bonanomi et al., 2020), such microbiome shifts may further contribute to the observed patterns of virus incidence.

### 4.2 DMDS efficacy against nematodes and associated viruses

Our results indicate a significant role of root-knot nematodes in shaping ToBRFV epidemiology in greenhouse tomatoes and that targeting nematode populations not only reduces root damage but may also indirectly limit viral spread, providing a dual benefit for crop health. Among available approaches, chemical fumigation remains a practical method for suppressing root-knot nematodes in intensive greenhouse systems, particularly where other control measures achieve only partial reduction (Loffredo et al., 2024; Pontes et al., 2024). Conventional nematicides are increasingly restricted due to environmental concerns (Sasanelli et al., 2021). Unlike methyl bromide, DMDS has zero ozone depletion potential (Heller et al., 2009) and represents a promising, safer fumigant alternative that has been widely evaluated for its effectiveness against nematodes (Yan et al., 2019). In the present study, DMDS demonstrated promising efficacy in controlling *Meloidogyne incognita*, with effective application rates as low as 30 g/m², although this provided only partial suppression of the associated virus. Maximal suppression of both the nematode and virus was achieved at higher doses of 60 g/m² DMDS. Several studies have reported that while DMDS is highly effective against nematodes at relatively low rates, its activity against fungal or oomycete pathogens is generally weaker and often requires higher doses, longer exposure periods, or combinations with other fumigants (Jin et al., 2016; Zanon et al., 2018; Yan et al., 2019). This differential sensitivity among soilborne organisms may be explained by fundamental differences in redox physiology. Endoparasitic nematodes are continually exposed to host-derived oxidative defenses and have therefore evolved highly efficient thiol-based antioxidant systems, including thioredoxin- and peroxiredoxin-dependent pathways, to detoxify reactive oxygen species. As a result, root-knot nematodes such as *Meloidogyne incognita* are considerably less sensitive to exogenous oxidative stress than free-living nematodes like *Caenorhabditis elegans* (Goverse and Smant, 2014). Disruption of these thiol-based redox systems compromises ROS scavenging capacity and leads to uncontrolled oxidative stress (Rhee et al., 2005; Lu and Holmgren, 2014; Ulrich and Jakob, 2019). Accordingly, DMDS, through its thiol-reactive disulfide chemistry, is thought to exert nematicidal activity by overwhelming antioxidant defenses and promoting lethal ROS accumulation (Ayaz et al., 2021; Wang et al., 2023). For instance, Yan et al. (2019) confirmed the efficacy of DMDS as a soil fumigant against *Meloidogyne* spp., with treatments ranging from 10 to 100 g/m² achieving 80–94% nematode suppression in tomato and other crops. Combinations of DMDS with other fumigants have further enhanced its effectiveness; for example, 1,3-dichloropropene (10 g/m²) plus DMDS (30 g/m²) significantly controlled *M. incognita* and suppressed *Fusarium oxysporum* and *Phytophthora* spp., outperforming DMDS alone (Jin et al., 2016). Similarly, reduced-dose strategies combining DMDS with metam sodium have produced synergistic suppression of nematodes, fungi, and weeds in cucumber field trials (Mao et al., 2019). Additional studies have reported DMDS activity against soil-borne fungi and disease complexes at rates of 30–60 g/m² (Pecchia et al., 2017; Ilieva et al., 2021). DMDS has also been reported to promote plant growth and exhibit antifungal activity against important plant pathogens such as *Sclerotinia* spp (Tyagi et al., 2020). Beyond plant pathogens, sulfur-containing volatiles including allyl methyl disulfide and dimethyl trisulfide (DMTS) exhibit strong antibacterial activity against human pathogens such as *Escherichia coli O157:H7* (Wu et al., 2023). Although direct evidence for DMDS activity against systemic plant viruses is limited, sulfur volatiles have been reported to reduce viral infectivity in animal systems (Ming et al., 2021; Mösbauer et al., 2021). In plants, garlic extracts, which naturally contain complex mixtures of sulfur compounds, including DMDS, have shown broad-spectrum antiviral activity, including against ToBRFV (Rouf et al., 2020; Iobbi et al., 2023). However, the specific compound(s) responsible for this antiviral activity have not been fully identified, and a direct link to DMDS remains unconfirmed. These effects are more likely driven by synergistic or additive interactions among multiple sulfur volatiles rather than by DMDS alone. Consequently, the reductions in virus incidence observed in our study are more plausibly attributed to nematode suppression and/or activation of plant defense mechanisms rather than a direct viricidal effect of DMDS. Nevertheless, the potential direct antiviral properties of DMDS in plants remain largely unexplored and warrant further detailed investigation. Taken together, these findings support the interpretation that nematode suppression—and the associated improvement in root health—is linked to reduced ToBRFV incidence under commercial greenhouse conditions. However, the relationships observed here should be interpreted as indirect and correlative, reflecting co-variation along a gradient of nematode pressure rather than a demonstrated causal pathway.

### 4.3 Implications for integrated greenhouse management

DMDS evaporates readily from soil due to its high vapor pressure at ambient temperature, which enhances its fumigant efficiency but raises concerns regarding emissions and environmental safety (Bhadra et al., 2021). To mitigate these effects, emission-reduction strategies have been developed, including sealing the soil surface with plastic films during fumigation followed by controlled aeration (Gao et al., 2011). These approaches effectively reduce emissions while maintaining high fumigation efficacy. When applied under such conditions, DMDS and its formulations have been shown in both greenhouse and field trials to suppress a wide range of soil-borne pests, including *Fusarium* spp., *Phytophthora* spp., root-knot nematodes (*Meloidogyne* spp.), *Sclerotinia minor*, *Verticillium dahliae*, *Rhizoctonia solani*, and *Pythium aphanidermatum* (Gómez-Tenorio et al., 2015; Huang et al., 2018; Mao et al., 2019; Yan et al., 2019; Tyagi et al., 2020). In our experiment, DMDS application at both low and high concentrations exhibited minimal or no phytotoxic effects on tomato plants while effectively controlling *M. incognita*. This selective efficacy may be partly explained by oxidative stress mechanisms, as previously discussed, although plants likely counteract potential oxidative damage through activation of antioxidant defense systems (Cannea and Padiglia, 2025). The low phytotoxicity of DMDS is further supported by its natural plant-derived origin and its pre-planting application mode, as the compound volatilizes and degrades rapidly in soil, minimizing the risk of residue accumulation in edible plant parts. Residue studies have confirmed that DMDS dissipates quickly, with final residues in cucumbers below 0.05 mg/kg—well under the established maximum residue limits (MRLs; Kaium et al., 2019). Similarly, in our tomato trials, DMDS residues remained below MRLs, confirming its safety for food production (unpublished data).

## 5 Conclusions

The frequent co-occurrence of root-knot nematodes (*Meloidogyne incognita*) and viral infections such as ToBRFV highlights the importance of managing nematode populations within integrated disease management strategies for greenhouse tomato production. Under the conditions examined in this study, higher levels of nematode-associated root damage were consistently associated with increased ToBRFV incidence across plots. In this context, DMDS functioned as an effective nematicidal treatment, generating a gradient of nematode suppression and corresponding variation in root health. Reductions in ToBRFV incidence observed in plots receiving higher DMDS application rates are therefore interpreted as indirect and associative, reflecting improved root condition and altered soil–plant interactions rather than a direct antiviral effect of the fumigant. By suppressing nematode populations and modifying the soil environment, DMDS may contribute to conditions that are less favorable for the development of complex, multi-pathogen disease scenarios in intensive greenhouse systems. These findings support the potential role of nematode management as a component of integrated disease management programs aimed at mitigating disease complexes under protected cultivation. Future research should further explore the interactions among soil microbiomes, nematode activity, and viral epidemiology, as well as the indirect pathways through which nematode suppression influences viral occurrence in tomato.

## Data Availability

The original contributions presented in the study are included in the article/supplementary material. Further inquiries can be directed to the corresponding authors.
